# Sitagliptin elevates plasma and CSF incretin levels following oral administration to nonhuman primates: relevance for neurodegenerative disorders

**DOI:** 10.1007/s11357-024-01120-4

**Published:** 2024-03-27

**Authors:** Yazhou Li, Kelli L. Vaughan, Yun Wang, Seong-Jin Yu, Eun-Kyung Bae, Ian A. Tamargo, Katherine O. Kopp, David Tweedie, Cheng-Chuan Chiang, Keith T. Schmidt, Debomoy K. Lahiri, Michael A. Tones, Margaret M. Zaleska, Barry J. Hoffer, Julie A. Mattison, Nigel H. Greig

**Affiliations:** 1 Translational Gerontology Branch, National Institute On Aging, Intramural Research Program, National Institutes of Health, Baltimore, MD 21224 USA; 2 Center for Neuropsychiatric Research, National Health Research Institutes, Zhunan, Taiwan 35053; 3 Department of Physical Medicine and Rehabilitation, Johns Hopkins University School of Medicine, Baltimore, MD 21287 USA; 4 Clinical Pharmacology Program, Center for Cancer Research, National Cancer Institute, National Institutes of Health, Bethesda, MD 20892 USA; 5 Departments of Psychiatry and Medical & Molecular Genetics, Indiana University School of Medicine, Indianapolis, IN 46202 USA; 6 Cadre Bioscience, Saint Louis, MO 63110 USA; 7 Neuro-D Consulting LLC, Penn Valley, PA 19072 USA; 8 Department of Neurosurgery, University Hospitals of Cleveland, Case Western Reserve University School of Medicine, Cleveland, OH 44106 USA

**Keywords:** Sitagliptin, Nonhuman primate, Parkinson’s disease, Glucagon-like peptide-1 (GLP-1), Glucose-dependent insulinotropic polypeptide (GIP), Dipeptidyl peptidase 4 (DPP-4)

## Abstract

The endogenous incretins glucagon-like peptide-1 (GLP-1) and glucose-dependent insulinotropic polypeptide (GIP) possess neurotrophic, neuroprotective, and anti-neuroinflammatory actions. The dipeptidyl peptidase 4 (DPP-4) inhibitor sitagliptin reduces degradation of endogenous GLP-1 and GIP, and, thereby, extends the circulation of these protective peptides. The current nonhuman primate (NHP) study evaluates whether human translational sitagliptin doses can elevate systemic and central nervous system (CNS) levels of GLP-1/GIP in naive, non-lesioned NHPs, in line with our prior rodent studies that demonstrated sitagliptin efficacy in preclinical models of Parkinson’s disease (PD). PD is an age-associated neurodegenerative disorder whose current treatment is inadequate. Repositioning of the well-tolerated and efficacious diabetes drug sitagliptin provides a rapid approach to add to the therapeutic armamentarium for PD. The pharmacokinetics and pharmacodynamics of 3 oral sitagliptin doses (5, 20, and 100 mg/kg), equivalent to the routine clinical dose, a tolerated higher clinical dose and a maximal dose in monkey, were evaluated. Peak plasma sitagliptin levels were aligned both with prior reports in humans administered equivalent doses and with those in rodents demonstrating reduction of PD associated neurodegeneration. Although CNS uptake of sitagliptin was low (cerebrospinal fluid (CSF)/plasma ratio 0.01), both plasma and CSF concentrations of GLP-1/GIP were elevated in line with efficacy in prior rodent PD studies. Additional cellular studies evaluating human SH-SY5Y and primary rat ventral mesencephalic cultures challenged with 6-hydroxydopamine, established cellular models of PD, demonstrated that joint treatment with GLP-1 + GIP mitigated cell death, particularly when combined with DPP-4 inhibition to maintain incretin levels. In conclusion, this study provides a supportive translational step towards the clinical evaluation of sitagliptin in PD and other neurodegenerative disorders for which aging, similarly, is the greatest risk factor.

## Introduction

A link between neurodegenerative disorders, particularly Parkinson’s disease (PD), and type 2 diabetes mellitus (T2DM) that, likewise, is a progressive age-associated degenerative disorder has been suggested for decades [1]. T2DM has been associated with an increase in the risk for PD as well as an exacerbation of the PD phenotype involving greater axial motor symptoms, such as gait disturbances and postural instability, in addition to cognitive impairment [1–5]. A number of common risk factors have been suggested to underlie the development of T2DM and PD. In addition to age, these include inflammation, oxidative stress, and insulin deficiency or resistance [6, 7]. Should this be true, it is feasible that drugs effective in treating one disorder may have activity in the other, thereby supporting the repurposing of drugs of known tolerability and efficacy. The repurposing of existing FDA-approved, well-tolerated clinically used drugs provides an efficient, rapid, and cost-effective approach to develop drug candidates for ineffectively treated disorders that potentially share common underlying mechanisms. Recent epidemiological data on individuals with T2DM prescribed antidiabetic drugs indicates that those taking either dipeptidyl peptidase-4 (DPP-4) inhibitors or glucagon-like peptide-1 (GLP-1) receptor (R) agonists were 36 to 60% less likely to develop PD, as compared to those on other antidiabetic medications, such as metformin or sulfonylureas [8].

Here, we evaluated the potential of a widely used DPP-4 inhibitor, also known as the gliptin drug class, as a new treatment strategy to mitigate dopamine (DA) neuron dysfunction and loss in PD. DPP-4 (EC 3.4.14.5), also known as CD26, is a S9B member of the proloyl oligopeptidase family of related glycoproteins. Among its structural regions, DPP-4 possesses a catalytic domain with the ability to cleave dipeptides from the N‐terminus of a series of physiologically relevant peptides that have a proline or alanine at their penultimate position. The two incretin hormones GLP-1 and glucose-dependent insulinotropic polypeptide (GIP) possess an alanine as their second amino acid. Hence, their two N-terminal amino acids are cleaved by DPP-4 to, thereby, virtually inactivate their insulin-stimulating actions [9]. This results in a short circulating half-life (T_1/2_) for active (intact) GLP-1 and GIP. DPP-4 is widely expressed across cell types and organs. Its inhibition results in elevated levels of both incretins that, in turn, results in the lowering of circulating glucose levels consequent to stimulation of pancreatic β-cell insulin secretion and inhibition of glucagon secretion [9–11]. GLP-1 and GIP additionally have trophic and protective actions that, like their insulinotropic actions, are mediated via their G-protein-coupled receptors [9, 11, 12]. These receptors, similarly, are widely expressed across cell types and organs, including within the brain [13, 14]. Elevated levels of GLP-1 and GIP or use of their DPP-4-resistant analogues has been shown to provide neurotrophic, neuroprotective, and anti-inflammatory actions in cellular and animal models of PD as well as other age-associated neurodegenerative disorders [6, 13–28]. This has spurred the evaluation of the long-acting GLP-1 receptor agonist Exenatide, a peptide drug approved and efficacious in T2DM, as a new treatment approach in human PD clinical trials, where it has demonstrated promising efficacy [29–32].

DPP-4 inhibition hence provides an alternative strategy to the use of direct GLP-1 and/or GIP receptor agonists to stimulate incretin receptors within the brain as a treatment approach for PD. Elevated systemic incretin levels should augment levels within the brain, as incretins have been shown to cross the blood–brain barrier [33–35]. Furthermore, gliptins are well tolerated, clinically approved worldwide for the effective treatment of T2DM and, importantly, have glucose-dependent actions and thus can be administered to euglycemic subjects without the risk of hypoglycemia [36–41]. Finally, DPP-4 inhibitors are oral drugs and present the potential benefit of jointly enhancing GLP-1 and GIP actions to impart synergistic actions [36–41].

We previously demonstrated in the rat that human clinically equivalent doses of the approved DPP-4 inhibitors sitagliptin and PF-00734,200 (gosogliptin) that induced in excess of 60% and 20% inhibition of plasma and brain DPP-4 activity, respectively, mitigated the loss of dopaminergic neurons and dopamine levels, as well as methamphetamine-mediated rotation, in rats challenged with a unilateral medial forebrain bundle 6-hydroxydopamine (6-OHDA) lesion [42]—a classical rodent model of PD. This gliptin-induced inhibition of DPP-4 activity led to a rise in systemic and brain GLP-1 and GIP levels in rats [42], and supports the prospective repurposing of either gliptin as a potential PD treatment. In the present study, we evaluated whether clinically translatable doses of sitagliptin could, similarly, elevate GLP-1 and GIP plasma and brain levels in naive, non-lesioned nonhuman primates (NHPs) following DPP-4 inhibitory action—as NHPs, compared to rodent studies, provide a closer translational model to humans. We selected sitagliptin over PF-00734,200 to evaluate as the former (*Januvia*) is clinically approved and widely available worldwide. Specifically, our focus in this current study was to assess whether sitagliptin doses that provided the neuroprotective/regenerative and anti-inflammatory actions in a rat 6-hydroxydopamine (6-OHDA) model of PD [42] could be safely tolerated in NHPs and achieve parallel pharmacodynamic actions (i.e., alike drug-induced pharmacological effects). In particular, we focused on matching sitagliptin plasma concentrations (i.e., pharmacokinetics) in NHP to those safely achieved in prior human [41, 43, 44] and rat PD studies [42] as a means to support translation into a future human sitagliptin PD clinical trial. Our cellular studies in human SH-SY5Y cells and in rat primary dopaminergic neuronal cultures, herein, demonstrate that elevated incretin levels, particularly when combined with a DPP-4 inhibitor, provide neuroprotective activity.

## Methods

### Cellular studies

#### Human SH-SY5Y cell culture

Immortal undifferentiated SH-SY5Y human neuroblastoma cells (ATCC® CRL-22669™) and growth media were purchased from American Type Culture Collection (ATCC). Cells were used to a maximum of 15 passages, and were grown in a mixture of half Eagle’s Minimum Essential Medium (EMEM) (ATCC® 30–2003™) and half Ham’s F12-K (Kaighn’s) Medium (ATCC® 30–2004™) supplemented with 10% heat-inactivated fetal bovine serum (Gibco™ cat no. 10082147) and 100 U/mL penicillin/streptomycin (Gibco™ cat no. 15140148). Cells were split at a 1:3 ratio every 5 days using 0.05% trypsin and 0.53 mM ethylenediaminetetraacetic acid (EDTA) (Invitrogen cat no. AM9912).

SH-SY5Y cells were treated with GIP (10 nM) or GLP-1 (10 nM) or vehicle, with or without a DPP-4 inhibitor (PF-00734,200: 1 µM). Five minutes later, the cells were treated with vehicle or freshly prepared 6-OHDA (100 µM) for 2 h. GIP, GLP-1, and DPP-4 inhibitor or vehicle were added to the media again after three washes. The cells were ultimately fixed at 22 h after 6-OHDA treatment ended (i.e., at 24 h from initiation of the study) and were stained with TOPRO-3 (1:2500, for 1 h at 37°C). The cells were then washed in PBS for three times and, thereafter, were imaged using a Licor Odyssey IR scanner. Cell density in each well was analyzed by using Licor Odyssey In Cell Western analyses software v 3.0, as previously described [45]. In these cell culture studies, the DPP-4 inhibitor PF-00734,200 was used in preference to sitagliptin consequent to its greater aqueous solubility (pilot studies indicated alike efficacy in cell cultures).

In the current study, TOPRO-3 was used as a marker of cell number since it is highly specific for DNA, and intensely stains the cell nuclei [46]. In this regard, TOPRO-3 has been previously used to quantify cell density in SH-SY5Y cells [47, 48]. Notably, TOPRO-3 can potentially be used as a marker of cell death, which can be visualized as brighter spots on a lower fluorescent background. When TOPRO-3 has been used to label nuclei in fixed or permeabilized cultured SH-SY5Y cells, TOPRO-3-mediated near-infrared fluorescence was found mostly in non-apoptotic and in a few in apoptotic (expressing activated caspase 3) SH-SY5Y cells [49]. In our study, TOPRO-3 was applied to cells after fixation with 4% PFA. The cells were washed in PBS for three times and, thereafter, were imaged using a Licor Odyssey IR scanner. Under these conditions, it is likely that any dead cells were washed away before quantifying fluorescence.

#### Primary cultures of rat ventral mesencephalon

Primary cultures were generated from embryonic (E14-15) ventral mesencephalon (VM) brain tissue removed from fetuses of timed-pregnant Sprague–Dawley rats (Charles River Laboratories, Wilmington, MA), in line with published procedures with minor modifications [24]. Briefly, the whole brain was removed aseptically, and a small piece of tissue containing the VM was separated. Blood vessels and meninges were detached, and remaining pooled VM tissues were gently trypsinized (0.25% (Invitrogen, Carlsbad, CA), 15 min at 37°C). Trypsin was removed with pre-warmed DMEM/F-12 (Invitrogen), and cells were dissociated by trituration and then counted and plated into 96-well (6.0 × 10^4^/well) cell culture plates that were pre-coated with poly-d-lysine (Sigma-Aldrich). The culture plating medium was Dulbecco’s modified Eagle medium/F12 supplemented with 10% heat-inactivated fetal bovine serum, 1 mM l-glutamine, and 2% B27 (Invitrogen). VM cells were maintained at 37°C in a humidified atmosphere (5% CO_2_ and 95% air) and fed by exchanging 50% of media with feed media (Neurobasal medium, Invitrogen) with 0.5 mM l-glutamate and 2% B27 with antioxidants supplement on DIV (days in vitro) 3 and 5. On DIV7, VM cultures were fed with media containing B27 supplement minus antioxidants (Invitrogen). Thereafter, freshly prepared 6-OHDA (100 µM in 20 µM ascorbic acid saline solution) or saline (with 20 µM ascorbic acid) was added to the wells on DIV 10. Following 2-h incubation, cultures were washed with feed media plus ( −) AO B27 three times. DPP-4 inhibitor (PF-00734,200), incretins, or vehicle was added to the well at the last wash as a post-treatment strategy. Cells were returned to a 37°C incubator for 22 h and then fixed with 4% paraformaldehyde (PFA) for immunoreactivity evaluation.

#### Statistics for in vitro studies

Values are expressed as means ± standard error of means (S.E.M) Student’s *t* test and 1- and 2-way ANOVA tests were used for statistical analysis. ANOVA on ranks was used when the normality assumption was violated. Post hoc Newman-Keuls test or Dunn’s test was used for all pairwise multiple comparisons. The Bonferroni correction was used for serial measurements. A statistically significant difference was defined as *p* < 0.05.

### Animal studies

#### Nonhuman primates

NHPs were evaluated in this study to provide a closer translational bridge to appraise the value of potentially repurposing sitagliptin as a PD treatment strategy in humans, to follow up on our prior study in rats [42]. Animals included *Macaca mulatta* of 7 to 20 kg weight, mixed gender, 12 to 19 years of age (*n* = 5 to 7 per group). Notably, these were non-lesioned NHPs that were maintained at the National Institutes of Health Animal Center (Poolesville, MD, USA) and housed in standard primate caging with a controlled temperature and humidity and a 12-h light cycle. Commercially prepared monkey chow was distributed twice per day along with daily food enrichment, and water was available ad libitum. Monkeys were observed daily for food consumption and overall well-being. Animal husbandry and all experimental procedures complied with the National Institutes of Health Guide for the Care and Use of Laboratory Animals (2011) and all procedures were approved by the National Institute on Aging’s Intramural Research Program Animal Care and Use Committee.

**Fig. 1:**
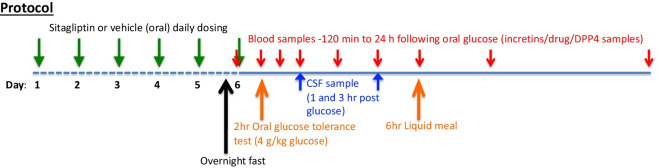
Nonhuman primate protocol. Vehicle or one of three doses of sitagliptin (5, 20, or 100 mg/kg) was administered orally once daily for 6 consecutive days to rhesus monkeys (7–20 kg weight, mixed gender, 12 to 19 years of age, *n* = 5 to 7 per group) to attain steady-state conditions prior to a classical oral glucose load, which was administered on the “test day” (day 6) to initiate endogenous incretin (GLP-1 and GIP) release, since incretin release from L and K cells is stimulated by food presence within the gastrointestinal tract. Time-dependent plasma and CSF samples were obtained to permit measurement of (i) GIP and GLP-1 levels, (ii) sitagliptin levels, and (iii) DPP-4 activity. Notably, a 6-h liquid meal was administered to the sedated monkeys to preserve blood glucose levels since all animals were fasted overnight prior to experimental day 6. Drug/vehicle administration was initiated at the same time across all NHPs, and thus plasma and CSF collection times were the same across animals

Specifically, in line with our prior rodent studies [42], we evaluated sitagliptin in NHPs to assess whether translational doses could elevate systemic (plasma) and central nervous system (CNS) incretin levels in a manner similar to that achieved in rats. Monkeys were evaluated and orally administered either vehicle or sitagliptin via orogastric tubing under light anesthesia once daily at the same time of day across all animals for 5 consecutive days prior to the experimental day in order to achieve a steady-state drug action on day 6, when blood and CSF samples were time-dependently collected (Fig. 1) to allow quantification of markers of drug concentration and action (i.e., GLP-1 and GIP levels, sitagliptin concentration, and DPP-4 activity). Three sitagliptin oral doses were evaluated under steady-state conditions (5, 20, and 100 mg/kg), with the two lower doses, importantly, being both of (i) direct human translational relevance, and (ii) direct translational relevance to our prior rat studies that demonstrated the ability of sitagliptin to mitigate loss of dopamine and dopaminergic cells in the striatum and substantia nigra of 6-OHDA challenged rats [42].

Specifically, the sitagliptin doses of 5 mg/kg, 20 mg/kg, and 100 mg/kg were administered once daily orally by orogastric gavage, and evaluated vs. a placebo (vehicle) control group.

**Fig. 2:**
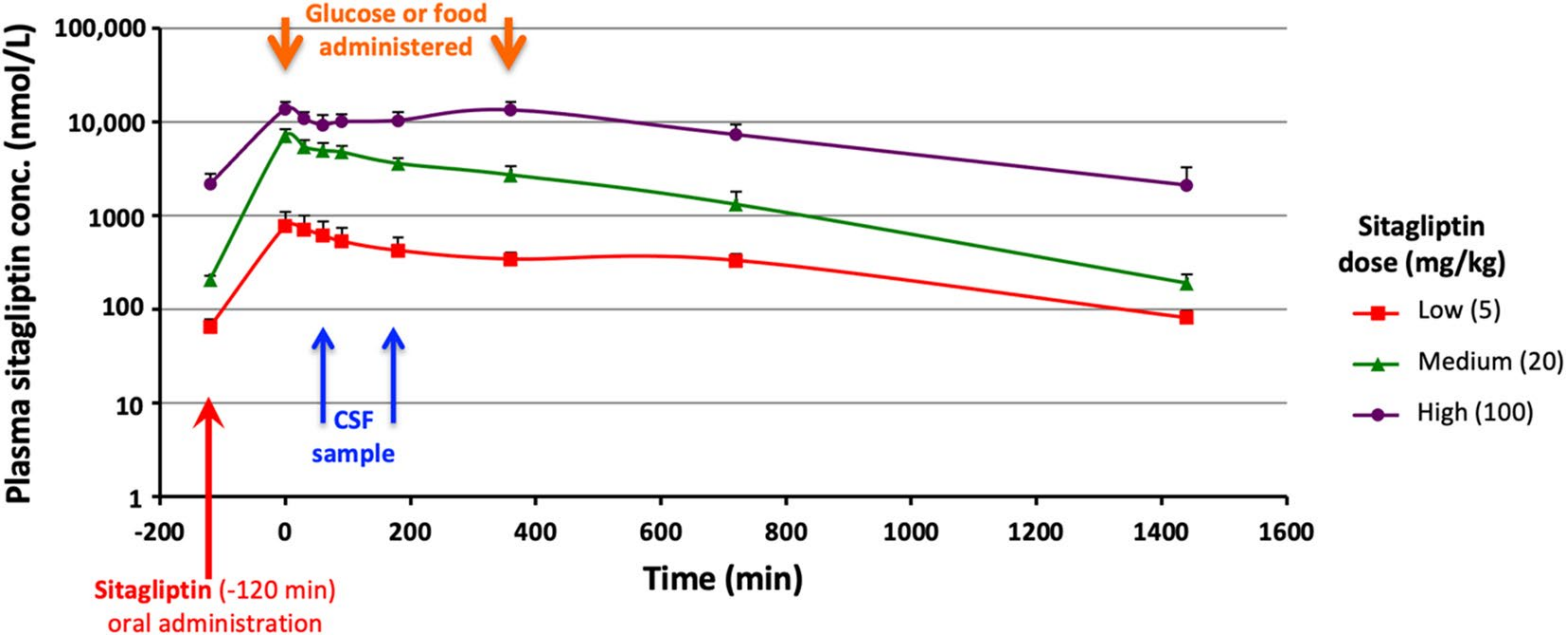
Plasma dose-dependent sitagliptin pharmacokinetics in nonhuman primates. Time-dependent plasma concentrations of sitagliptin following oral administration of one of three separate doses administered once daily (sitagliptin low 5 mg/kg, medium 20 mg/kg, high 100 mg/kg) were measured by LC/MS [43]. As noted, sitagliptin was administered at − 120 min (where zero represents the time when the oral glucose load was administered to initiate incretin release). Peak sitagliptin levels in plasma occurred at approx. 2 h post administration and were dose-dependent. All values are mean ± SEM

On the experimental day (day 6), following an overnight fast, animals were sedated for a baseline blood sample via an inserted indwelling saphenous vein catheter, followed by oral gavage of sitagliptin/placebo treatment. At 2-h post-drug/placebo dosing, the reported peak time of sitagliptin action [43, 44], blood glucose values were quantified from a blood sample obtained via toe prick using an Ascensia® Breeze 2 blood glucose monitoring system (Bayer HealthCare LLC., Mishawaka, IN). Immediately thereafter, an oral glucose tolerance test was performed to stimulate incretin secretion. This is designated as time zero (i.e., 0) in Figs. 2, 4 and 5. Specifically, a 4-g/kg glucose solution was administered orally by orogastric tubing, and blood samples were then time-dependently collected via the indwelling catheter (times 0, 30, 60, 90 min, and 3, 6, 12, and 24 h). CSF samples (cervical or lumbar by acute puncture) were drawn at 1 and 3 h post glucose administration (equivalent to 3 and 5 h following sitagliptin dosing), and a liquid meal was given at 6 h (Fig. 1).

### Assays

#### Measurement of incretins, DPP-4 activity, and sitagliptin levels in plasma and CSF

A DPP-4 inhibitor (PF-00734,200) was pre-added to fresh plasma and CSF samples to stabilize them for subsequent incretin assays; additional samples were flash frozen for later analysis. Further samples were collected without the inhibitor for DPP-4 analysis to permit quantification of DPP-4 inhibition. Incretin levels were measured with the following assays: Active GLP-1 ((ver 2 kit) Meso Scale Diagnostic), Active GIP (ELISA kit—IBL America), DPP-4 activity was evaluated by fluorometric assay using a DPPIV/CD26 assay kit for biological samples ((BML-AK498-0001) Enzo Life Sciences, Farmingdale, NY). In addition, separate plasma and CSF samples were flash frozen to allow evaluation of sitagliptin levels (pharmacokinetic analysis) by LC–MS utilizing the method of Zeng et al. [50].

### Statistics for pharmacokinetic studies

Values are expressed as means ± S.E.M. Kolmogorov–Smirnov test was used to examine the normality. Student’s *t* test and 1- and 2-way ANOVA tests were used for statistical analysis. ANOVA on ranks was used when the normality assumption was violated. Post hoc Newman-Keuls test or Dunn’s test was used for all pairwise multiple comparisons. The Bonferroni correction was used for several measurements. A statistically significant difference was defined as *p* < 0.05.

A noncompartmental approach to pharmacokinetic analysis was employed using Phoenix WinNonlin v8.3 (Certara Corp, Cary, NC). The maximum plasma concentration (*C*_max_) and the time of maximum plasma concentration (*T*_max_) were recorded as observed values. The area under the concentration–time curve (AUC) from time zero to the time of the final quantifiable sample (AUC_last_) was calculated using the linear-up/log-down trapezoidal method (model type Plasma (200–202)). AUC_INF_ (the AUC from time zero to infinity) was calculated by extrapolation by dividing C_last_ (the last measurable drug concentration) by the rate constant of the terminal phase, l_Z_ (Kel). This constant was determined from the slope of the terminal phase of the concentration–time curve using uniformly weighted least squares as the estimation procedure and acceptance criteria of (i) adjusted *r*^2^ > 0.8 and (ii) includes > 3 time points in the terminal phase.

## Results

### Nonhuman primate studies

#### Rationale

Our prior evaluation of clinically translatable oral doses of gliptins in 6-OHDA-induced Parkinsonian rats demonstrated mitigation of dopaminergic cell loss, dopamine levels, and motor impairment [42]. This efficacy associated with an inhibition of DPP-4 activity and a rise in GLP-1 and GIP levels in plasma and brain [42], and was achieved with a gliptin dose that provided a *C*_max_ plasma concentration of 976 nmol/L [42] that matched those reported in humans administered a routine clinical sitagliptin dose [41, 43, 44], thereby supporting consideration of a gliptin as a treatment for human PD. In the light of these prior rodent studies, we herein evaluated sitagliptin in healthy NHPs as a translational step in a closer animal species to humans to further evaluate repurposing sitagliptin for PD. Our focus was to see whether clinically translatable sitagliptin doses in NHPs (that achieve similar plasma concentrations to our prior rat study and those reported in humans [41–44]) could provide elevations in GLP-1 and GIP levels, and an decline in DPP-4 activity that associated with the efficacy of sitagliptin in our 6-OHDA challenged rat study [42]. Hence, in the present NHP study, sitagliptin or vehicle was administered orally once daily for 5 consecutive days to achieve steady-state drug action and, on experimental day 6, the drug’s time-dependent pharmacokinetics and pharmacodynamics were then evaluated (Fig. 1).

The sitagliptin doses of 5 mg/kg, 20 mg/kg, and 100 mg/kg were administered once daily orally by orogastric gavage, and evaluated vs. a placebo (vehicle) control group. These sitagliptin doses were chosen as (i) the 5 mg/kg (low dose) is equivalent to 100 mg in a 65-kg human normalized to body surface area, in accord with FDA guidelines [51], which is the routine human sitagliptin dose in T2DM [41] that, additionally, is well tolerated in the elderly [52]. This dose, importantly, also translates to the dose of sitagliptin (10 mg/kg in rat) that demonstrated neuroprotective actions when administered 7 days prior to a 6-OHDA lesion [42]. (ii) The sitagliptin 20 mg/kg (medium dose) is equivalent to 400 mg in a 65-kg human normalized to body surface area. This represents a daily human dose safely used in prior sitagliptin clinical trials [43] and, notably, also translates to the dose of sitagliptin (30 mg/kg in rat) that demonstrated neuroregenerative efficacy against a 6-OHDA lesion in our prior rodent study [42]. (iii) The 100 mg/kg (high dose) is the noted maximally well-tolerated sitagliptin dose in NHPs from Merck’s data on file (see Reference 33 within [44]). It is not a dose that can be effectively translated into humans.

#### Tolerability

In a pilot study, rhesus monkeys found the taste of sitagliptin aversive and refused to voluntarily take the drug. As a consequence, the agent or vehicle was administered via orogastric gavage under light sedation. Each of the three doses of sitagliptin proved to be well-tolerated when administered daily during our 6-day study, and were not associated with obvious clinical aberrant effects, which is in accord with prior human and NHP studies [43, 53].

#### Pharmacokinetics

NHPs dosed orally with sitagliptin demonstrated readily detectable drug levels in plasma. As evident in Fig. 2, following 5-day consecutive once daily oral dosing, a 24-h resting level of sitagliptin was present at the time of dosing on day 6 (− 120 min; 5 mg, 65 nmol/L; 20 mg, 208 nmol/L; 100 mg, 2183 nmol/L), and plasma drug levels remained above the detectable limit throughout the 24-h collection period of the study for all sitagliptin doses. Peak plasma sitagliptin concentrations (*C*_max_) were achieved at 120 min (*T*_max_) post oral drug administration across doses (Fig. 2). *C*_max_ values are listed in Table 1, and coincided with zero time in relation to administration of an oral glucose load (Fig. 1) that, to re-emphasize, was administered to prompt incretin release from gastrointestinal L and K cells. Plasma sitagliptin concentrations proved to be dose-dependent, and the 5- and 20-mg/kg doses declined monophasically. In contrast, time-dependent plasma levels achieved with the high 100 mg/kg dose appeared to be maintained between the 2- and 8-h sampling times and, thereafter, declined monophasically (Fig. 2). Peak plasma levels achieved following 5 mg and 20 mg sitagliptin dosing compared favorably with those reported for the human clinical doses of 100 and 400 mg, respectively (Table 1).

**Table 1:** Comparison between nonhuman primate *C*_max_, *T*_max_, and *T*_1/2_ values of 5, 20, and 100 mg/kg oral sitagliptin and values reported in humans and rats for the equivalent dose

NHP sitagliptin dose	NHP *C*_max_	NHP PK parameter	FDA equivalent dose	Comparative *C*_max_	Human PK parameter
5 mg/kg	768.1 nmol/L	*T*_max_ 2 h *T*_1/2_ 8.4 h	Human: 100 mg Rat: 10 mg/kg	Human: 817 nmol/L [54] 959 nmol/L [55] 747 nmol/L [43] Rat: 976 nmol/L [42]	Human: *T*_max_ 1–3 h *T*_1/2_ 11–13 h
20 mg/kg	7060.7 nmol/L	*T*_max_ 2 h *T*_1/2_ 5.0 h	Human: 400 mg	Human: 5000 nmol/L [43]	
100 mg/kg	13,650.9 nmol/L	*T*_max_ 2–8 h	No equivalent human dose		

Select pharmacokinetic values of three doses of sitagliptin administered orally once daily to NHPs (5, 20, and 10 mg/kg) and comparison to equivalent translational doses (100 and 400 mg) reported in humans and to 10 mg/kg in the rat

*C*_max_ peak plasma concentration, *T*_max_ time to peak plasma concentration, *T*_*1/2*_ half-life (i.e., time required for plasma drug concentration to decrease by 50%)

Shown in Table 2, there is an approximate tenfold increase in *C*_max_ between the sitagliptin 5 mg/kg and 20 mg/kg doses in NHP and only a ~ twofold increase between the 20 mg/kg and 100 mg/kg dose. For AUC_inf_, an approximate sixfold increase was found between the 5 mg/kg and 20 mg/kg dose, and a ~ fourfold increase between the 20 mg/kg and 100 mg/kg dose. This is consistent with the plateau evident with the concentration–time curve of the 100 mg/kg dose (Fig. 2). In this light, it is likely that the absorption of sitagliptin is saturable in NHPs, with the 100 mg/kg dose being well above what the gastrointestinal tract of the primate can absorb, and, if correct, substantial drug would not reach the circulation. Of note, absorption of sitagliptin was not observed to be saturable in a prior study in rats (dosed between 2 and 180 mg/kg) [56].

**Table 2:** Pharmacokinetic parameters of 5, 20, and 100 mg/kg sitagliptin in nonhuman primates

Dose	Kel (1/h)	Half-life (h)	*T*_max_ (h)	*C*_max_ (nmol/L)	*T*_last_ (h)	*C*_last_ (nmol/L)	AUC_last_ (h* nmol/L)	AUC_INF__obs (h* nmol/L)
5 mg/kg	0.08212	8.4	2	768.1	26	81.0	7826.7	8813.5
20 mg/kg	0.13927	5.0	2	7060.7	26	190.5	49,560.6	50,928.2
100 mg/kg	0.10262	6.7	2	13,650.9	26	2113.6	193,023.4	213,620.6

Dose-dependent pharmacokinetic values of sitagliptin administered orally once daily to NHPs were determined by noncompartmental data analysis using Phoenix WinNonlin v8.3 (Certara Corp, Cary, NC). The elimination rate constant (Kel) represents the fraction of drug eliminated per unit of time. *T*_last_ and *C*_last_ are the time and concentration of the last sampling of sitagliptin. AUC_last_ represents the area under the concentration–time curve to the last measurable sample (26 h across doses) and AUC_INF__obs is the AUC extrapolated to infinity

**Fig. 3:**
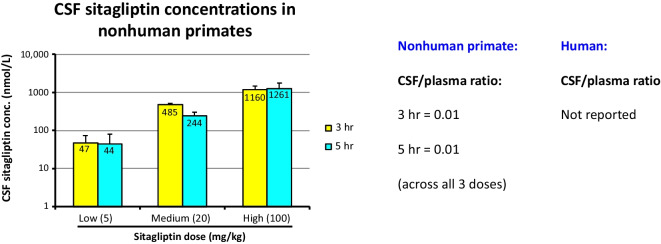
CSF sitagliptin levels in nonhuman primates. CSF dose-dependent sitagliptin pharmacokinetics in NHPs and CSF/plasma concentration ratio were obtained at 3 and 5 h post sitagliptin oral administration. All values are mean ± SEM

Sitagliptin levels in CSF have not been previously reported in NHPs or humans. Figure 3 shows that levels were detectable in CSF at 3 and 5 h following sitagliptin administration to monkeys (i.e., 1 and 3 h following the oral glucose load), and were dose-dependent. The CSF/plasma sitagliptin concentration ratio was 0.01, as evaluated across the 3 doses at 3 h post drug administration, and, likewise, was 0.01 as similarly evaluated at 5 h post sitagliptin administration.

#### Pharmacodynamics

**Fig. 4:**
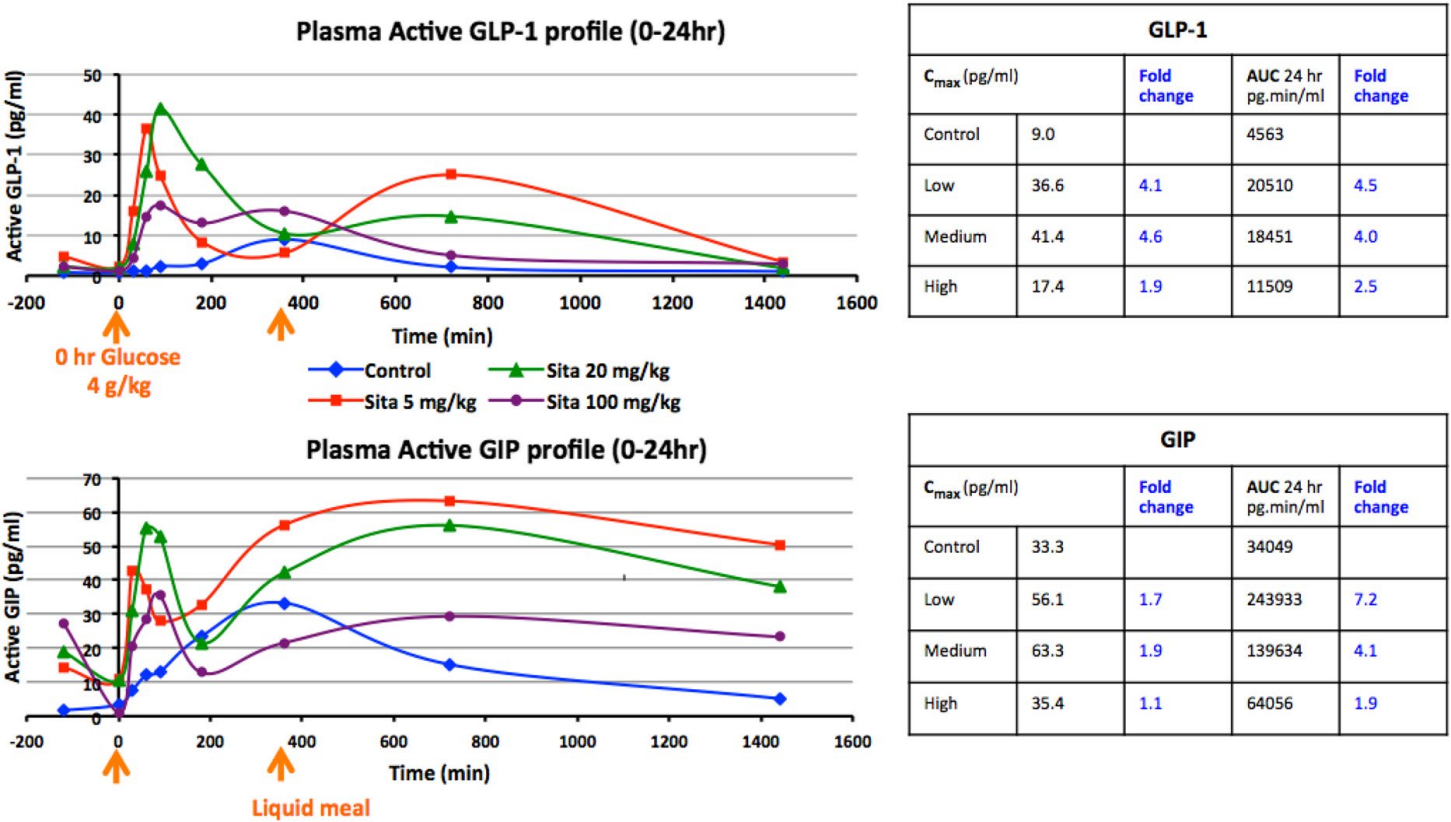
Time-dependent changes in GLP-1 and GIP levels following sitagliptin administration in nonhuman primates. Sitagliptin elevates plasma incretin levels in NHPs. *Top:* time- and dose-dependent levels of GLP-1. *Bottom:* similarly, of GIP in plasma following administration of vehicle or sitagliptin (5, 20, or 100 mg/kg P.O., at − 120 min), an oral glucose load (4 g/kg) at zero time and a liquid meal (6 h). Values are means*.* N.B. Zero time (0 min) is designated as the time of oral glucose administration to initiate incretin release, and sitagliptin was administered 120 min prior to this. A liquid meal was administered at 6 h to maintain the animals following an overnight fast

**Fig. 5:**
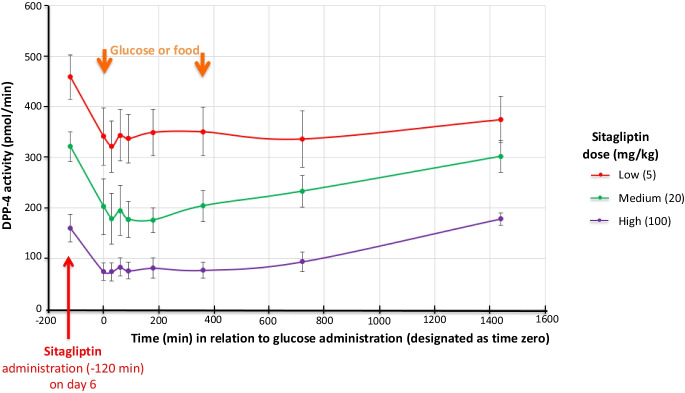
Time-dependent changes in plasma DPP-4 activity following sitagliptin administration in nonhuman primates. Sitagliptin time- and dose-dependently reduces (i.e., inhibits) plasma DPP-4 activity (pmol/min). Values are mean ± SEM*.* DPP-4 activity changed < 5% across timepoints in animals administered vehicle alone (i.e., 0 mg sitagliptin)—not shown. N.B. The zero-time point (0 min) is designated as the time of glucose administration to initiate incretin release

Time-dependent plasma concentrations of both GLP-1 and GIP were markedly elevated in sitagliptin-treated groups compared to vehicle-treated (control) animals across almost all times evaluated (Fig. 4). Elevations were significant and greatest for the clinically translatable 5- and 20-mg/kg sitagliptin doses. Dose-dependence was not evident with the high sitagliptin (100 mg/kg) dose, suggestive of induction of a compensatory mechanism during the 6-day dosing period of this maximal non-translatable dose in monkey. Consequent to the glucose load (time zero) and administration of a liquid meal at 6 h, administered in view of the animals being fasted over the previous night, two peaks in plasma incretin levels were evident that occurred at 30 to 60 min and at approximately 12 h. The clinically translatable 5 mg/kg and 20 mg/kg sitagliptin doses elevated peak plasma GIP and GLP-1 levels by 2- to fivefold over those achieved in control, vehicle administered, animals. The area under the time-dependent concentration curves was elevated by some 4.0- to sevenfold. Sitagliptin induced a dose- and time-dependent reduction in DPP-4 activity. As sitagliptin was administered daily over 5 subsequent days to achieve a steady-state drug action prior to sampling on day 6 administration, residual DPP-4 inhibition was evident at the time of sitagliptin dosing on day 6 remaining from the prior dose on day 5 (Fig. 5). Thereafter, DPP-4 activity levels declined and achieved a relatively steady-state reduction between the time of glucose administration, which is designated as time “0 min” at 120 min following sitagliptin dosing and 12 h (Fig. 5). A remaining reduction in DPP-4 activity was apparent in the 24-h plasma sample, in line with the sample taken at the time of sitagliptin dosing on day 6 which occurred 24 h following day 5 dosing. In contrast, in NHPs administered vehicle (i.e., control animals) DPP-4 activity varied less than 5% across time points.

**Fig. 6:**
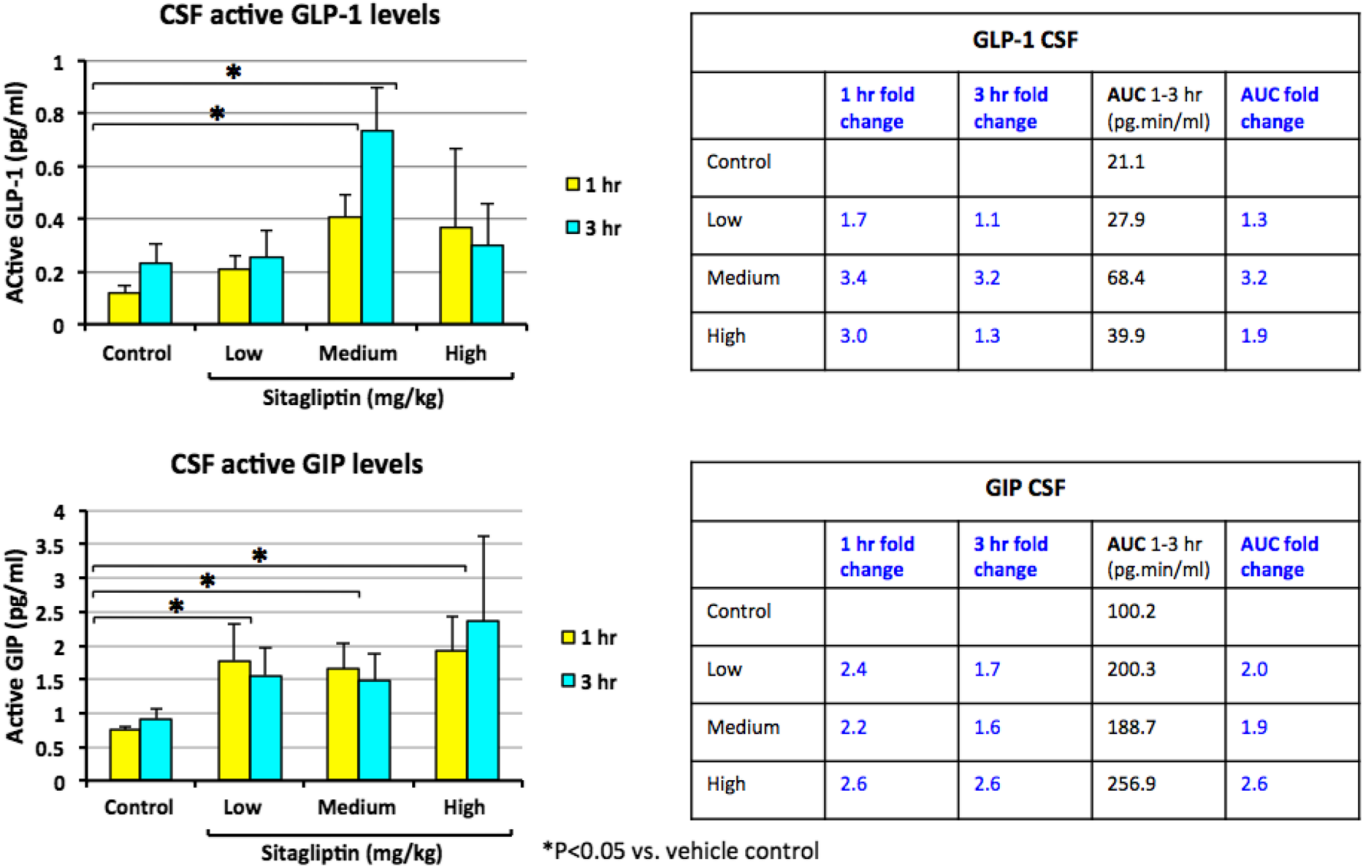
Time-dependent changes in incretin levels achieved in CSF following oral sitagliptin administration in nonhuman primates. Sitagliptin elevates CSF incretin levels in NHPs. *Top:* Time- and dose-dependent levels of GLP-1. *Bottom:* similarly, of GIP in CSF following oral administration of vehicle or sitagliptin (5, 20, or 100 mg/kg P.O.) at − 120 min, and a glucose load (4 g/kg) at zero time. The 1- and 3-h time points relate to the glucose load, and are at 3 and 5 h following sitagliptin dosing. Values are mean ± SEM (significant increase vs. vehicle *p < 0.05)

Incretin concentrations measured in CSF samples obtained at 3 and 5 h following day 6 sitagliptin and, notably at 1 and 3 h following glucose administration, are shown in Fig. 6. Parallel to that found in plasma, CSF levels of both GLP-1 and GIP were significantly elevated by 2.0- to 3.4-fold in sitagliptin vs. placebo-administered animals (Fig. 6).

### Cellular studies in immortal and primary neuron cultures

#### Protective action of gliptins + incretins in human SH-SY5Y cell culture

**Fig. 7:**
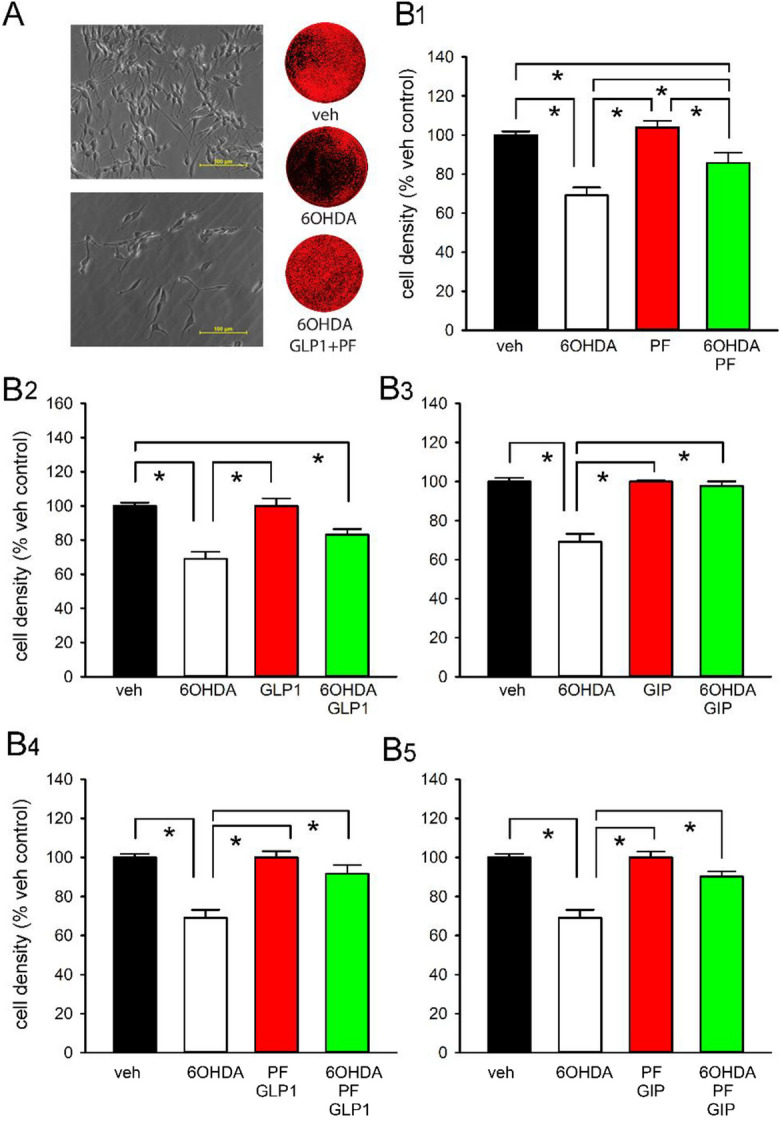
Combined DPP-4 inhibition and incretins mitigate 6‐OHDA–mediated cell loss in cultured SH-SY5Y neuronal cells. (**A**) Representative photomicrographs indicate that challenge with 100 µM 6‐OHDA (left lower panel) reduced cell density as compared to the control (left upper panel) (scale bar = 100 µm). (A, right panels) Cell nuclei were stained with TOPRO‐3 following 24-h drug treatment/6-OHDA challenge. Cell density across 96‐well plates was measured by a LiCor Odyssey image system. Three representative TOPRO‐3 images were obtained from cells administered vehicle, 6‐OHDA, and 6‐OHDA + GLP-1 + DPP-4 inhibitor (DPP-4I: PF-000734,200, 1 µM). (**B1**) TOPRO‐3 fluorescence density in each well was quantified. Challenge with 6‐OHDA (100 µM, 2 h) significantly reduced cell density (*p* < 0.001). Administration of (B1) DPP-4I (1 µM), (**B2**) GLP-1 (10 nM), or (B3) GIP (10 nM) ameliorated 6‐OHDA‐mediated cell loss. (**B3**) GIP alone or co‐administration of (B4) DDP-4I and GLP-1 or (B5) DDP-4I and GIP fully protected against cell loss, as no difference was found between these groups and the vehicle control group. All values are mean ± SEM. * *p* < 0.05, one-way ANOVA on Rank + Dunn’s test

Previous studies have demonstrated that human SH-SY5Y neuronal cells possess functional receptors for GLP-1 and GIP [23, 24, 57, 58]. To provide insight into whether elevations of the endogenous incretins, GIP and GLP-1, could prove valuable from a neurological perspective, cultured SH-SY5Y cells were treated with GIP (10 nM) or GLP-1 (10 nM) with or without a DPP-4 inhibitor (1 µM). Five minutes later, cells were exposed to vehicle or freshly prepared 6-OHDA (100 µM) for 2 h and then washed—to provide a pathological challenge. At 24 h after 6-OHDA challenge, cellular density was evaluated under light microscopy. 6-OHDA greatly reduced SH-SY5Y cell survival (Fig. 7(A), left panel), with TOPRO-3 fluorescence employed for quantifying cell density (Fig. 7(A), right panel). As shown in Fig. 7(B1), 6-OHDA challenge significantly reduced cell density (*p* < 0.001, one-way ANOVA). This was partially mitigated by DPP-4 inhibition (1 µM, Fig. 7(B1)) as well as by GLP-1 (10 nM, Fig. 7(B2), *p* < 0.05, one-way ANOVA on rank + Dunn’s test). Whereas DPP-4 inhibition or GLP-1, alone, did not fully block 6-OHDA-mediated degeneration (as a significant difference in cell density was found between 6-OHDA + DPP-4 inhibitor or 6-OHDA + GLP-1 and the without 6-OHDA vehicle controls (Fig. 7(B1, B2))), the co-administration of GLP-1 and DPP-4 inhibition fully prevented 6-OHDA-mediated cell loss. In this regard, the TOPRO fluorescence density was not different between the vehicle (without 6-OHDA challenge) and the GLP-1 + DPP-4 inhibitor + 6-OHDA groups (Fig. 7(B4)). GIP (10 nM) alone or together with a DPP-4 inhibitor also significantly antagonized 6-OHDA-induced cell loss (Fig. 7(B3, B5), *p* < 0.05, one-way ANOVA on rank + Dunn’s test).

#### Protective actions of gliptins + incretins in rat primary dopaminergic neuronal cultures

**Fig. 8:**
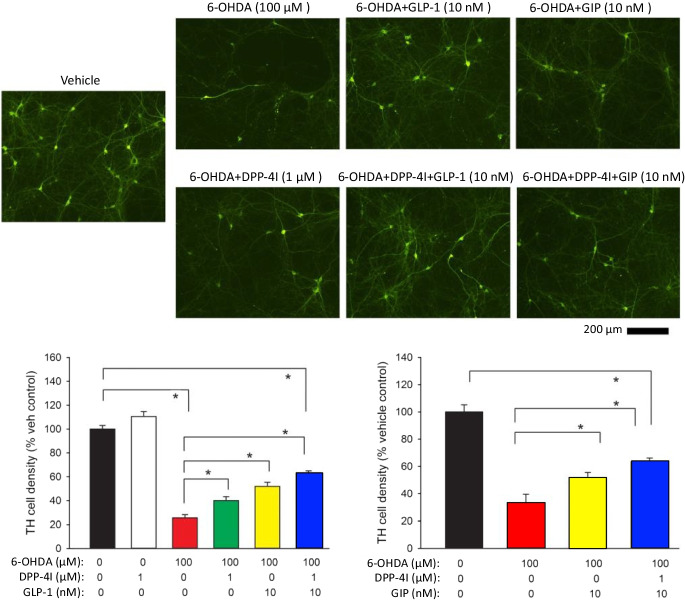
GLP‐1 and GIP provide neuroprotection against 6‐OHDA-induced toxicity in primary ventral mesencephalic cultures that is further augmented by DPP-4 inhibition. Primary cultures prepared from rat VM (E15) were challenged with 6‐OHDA (100 µM) for 2 h initiated 10 min following administration of a DPP-4 inhibitor (DPP-4I: PF‐00734,200 1 µM) with/without GLP‐1 or GIP (10 nM). Cells were fixed for tyrosine hydroxylase (TH) immunostaining at 22 h after washing. Treatment with GLP‐1 or GIP provided a significant amelioration of 6-OHDA-mediated toxicity. Co‐administration of DPP-4I further reduced 6‐OHDA-induced loss of TH cells. All values are mean ± SEM. *p < 0.05, one‐way ANOVA. All data were normalized to the mean of TH density in the vehicle (control) group

Similar to the responses found in immortal SH-SY5Y neuronal cells, challenge with 100 µM 6-OHDA significantly reduced TH ( +) cell density in rat primary ventromesencephalic neuronal cultures (Fig. 8), which provide a valuable translational model of functional dopaminergic neurons. GLP-1 (10 nM) or GIP (10 nM) partially but significantly mitigated the 6-OHDA-mediated reduction in TH cell density. This protective response of GIP and GLP-1 was significantly potentiated by co-administration of a DPP-4 inhibitor (PF-00734,200 1 µM).

Together, these neuronal cell culture studies suggest that GLP-1, GIP, and DPP-4 inhibition can mitigate the adverse action of 6-OHDA on cell survival, and that the combination of an incretin + DPP-4 inhibitor is particularly effective, and is in line with prior reports of the presence of DPP-4 activity in brain as well as neuronal cell lines [58–60].

## Discussion

Nonhuman primate pharmacokinetic/pharmacodynamic studies were undertaken to evaluate whether elevations in plasma and, more importantly, CNS levels of GIP and GLP-1 could be achieved with clinically translatable doses of the widely used DPP-4 inhibitor, sitagliptin, that were associated with neuroprotective, neuroregenerative, and anti-inflammatory actions that we demonstrated in a prior study involving a medial forebrain bundle 6-OHDA rat lesion model of PD [42]. In both our present NHP and prior rat studies, clinically translatable sitagliptin doses were selected to evaluate across species based on body surface area normalization, according to FDA guidelines to achieve equivalent dose selection for translation from animal to human studies [51].

In the present NHP study, our selected doses of 5 mg/kg and 20 mg/kg sitagliptin achieved similar peak plasma levels to their matched human doses of 100 mg and 400 mg once daily sitagliptin, which represent the routine human dose used in T2DM and a well-tolerated higher dose, respectively [41, 43, 44]. Notably, the peak mean plasma level of sitagliptin achieved in our NHPs, 768 nmol/L following 5 mg/kg sitagliptin, is in accord with peak mean plasma levels reported in humans administered the equivalent sitagliptin 100 mg dose (817 nmol/L [54], and 959 nmol/L [55]), thereby supporting the clinical relevance of our data. Key pharmacokinetic parameters of the time of maximal plasma levels (*T*_max_) and half-life of disappearance (*T*_1/2_), likewise, were well-matched across species (Table 1). These same NHP doses in our present study, notably, align with those selected in our previous rat 6-OHDA studies (10 and 30 mg/kg sitagliptin [42]) in which sitagliptin significantly elevated plasma and brain incretin levels, and mitigated neuroinflammation and, importantly, neurodegeneration of the dopaminergic system in a well characterized rodent PD model [42]. Specifically, the NHP and rat plasma concentrations of 768 and 976 nmol/L, respectively, closely align and, thereby, indicate that the selected doses we evaluated across species are, indeed, equivalent. Likewise, these measured rat plasma sitagliptin concentrations compare favorably to reported levels quantified in human pharmacokinetic studies following a routine sitagliptin clinical dose of 100 mg [54, 55]. Consequently, both from an allometric scaling and a pharmacokinetic perspective, the sitagliptin doses evaluated in our NHP and prior rodent studies fully align with approved doses that can be administered to humans.

Also notable, our NHP study demonstrates that these clinically achievable 5 mg/kg and 20 mg/kg daily sitagliptin doses were well-tolerated and significantly elevated plasma incretin levels by some 4.0- to sevenfold. CSF incretin levels were likewise elevated by some 2.0- to 3.4-fold over vehicle (control) values. These values are in line with elevations in CNS levels of incretins measured in our rodent studies that associated with neuroprotective and neuroregenerative actions in the 6-OHDA model of PD [42], and parallel-gliptin mediated elevations in endogenous incretins reported in humans [61]. Interestingly, the sitagliptin-mediated increases in brain incretins found in the present monkey study were achieved following administration of a gliptin that appears to reach minimal levels in the brain, possessing a CSF/plasma concentration ratio of 0.01 across all three evaluated doses (5, 20, and 100 mg/kg) and evaluated at two time points (3 and 5 h post sitagliptin dosing). To our knowledge, the quantitative brain entry of sitagliptin has not previously been reported, and our CSF/plasma ratio determined in monkey compares favorably with the 0.07 brain/plasma ratio found in rat [41, 43, 44]. Whereas sitagliptin has a reported log *P* value of 1.5 [62] that is considered favorable for brain entry [63], and its chemical structure complies with the Lipinski rule of 5 [64], sitagliptin has been reported to be a substrate for p-glycoprotein and organic anion transporter 3 (hOAT3) in relation to its renal clearance [65]. Such multi-drug resistance transporters are similarly present at the level of the blood–brain barrier, and associated with the efflux of drugs from the brain [66]. This may potentially, in part, account for sitagliptin’s low levels in the brain and CSF of rats and NHPs, respectively.

The reported DPP-4 IC_50_ value for sitagliptin is 18 nM [67], which was readily achievable in plasma in our monkey and rat pharmacokinetic studies and is potentially achievable in the brain, despite low brain uptake of sitagliptin. Futhermore, sitagliptin-mediated elevations in plasma GLP-1 and GIP may, in large part, account for the raised levels found in brain across our monkey and prior rodent studies, as these incretins are reported to cross the blood–brain barrier [33–35]. Of note, sitagliptin-induced elevations in brain levels of total GLP-1 and GIP have previously been reported by Gault et al. [68] in high-fat-fed mice, together with improvement in recognition memory, augmentation of hippocampal neurogenesis, and upregulation in the expression of several hippocampal genes. Genes of note included synaptophysin, sirtuin 1, glycogen synthase kinase 3β, superoxide dismutase 2, nuclear factor (erythroid-derived 2)-like 2, vascular endothelial growth factor, and, in particular, the receptors for GLP-1 and GIP.

Our prior quantification of brain GLP-1R and GIPR levels across age and in a 6-OHDA rodent model of PD, as well as in the substantia nigra of human PD and an age-matched control, demonstrates the retention of these drug targets during disease and aging [42]. In the light of multiple studies demonstrating the potential of both single and dual GLP-1 and GIP receptor agonists in mitigating neurodegenerative insults and neuroinflammation in preclinical animal models of neurological disorders as well as the efficacy of GLP-1 receptor agonists in human PD clinical trials [6, 13, 14, 26–29, 32, 69], the evaluation of DPP-4 inhibitors that, likewise, elevate plasma and brain GLP-1 and GIP levels is worthy of further consideration. In this regard, human epidemiological studies have demonstrated a reduced incidence of PD in subjects with T2DM taking a DPP-4 inhibitor [8, 70] and results from our cell culture studies, herein, are in line with numerous prior studies demonstrating that elevated incretin levels associate with neurotrophic and neuroprotective actions in a human immortal neuronal cell line and, importantly, in rat primary cultures rich in dopaminergic neurons [6, 13, 14, 26–29, 32, 71].

In conclusion, the present study, together with others [42, 72–76], provides a strong rationale for the future evaluation of a gliptin in PD. In this regard, we propose that sitagliptin be considered as a candidate for clinical trials in PD. Although its brain uptake is low (CSF/plasma ratio 0.01), its induced DPP-4 inhibition was sufficient to significantly elevate CNS incretin levels in line with levels in rodent studies associated with sitagliptin-mediated efficacy in a 6-OHDA model of PD [42]. To this end, a gliptin could be evaluated to augment the neurotrophic, neuroprotective, and anti-inflammatory actions of endogenous incretins either singularly, or in combination with an incretin mimetic. Although gliptins and incretin mimetics ultimately provide their pharmacological action through the same mechanism, the GLP-1/GIP receptors, their combination is not recommended in T2DM as it does not provide any clinically meaningful improvement in glycemic control over either agent singly [77, 78]. However, such additive or synergistic action might well be beneficial for a CNS-based disorder (in line with co-administration in Fig. 8). Importantly, sitagliptin is clinically approved, widely used, and well tolerated with weight neutral actions and no risk of hypoglycemia [39, 52, 53], which would allow rapid repurposing to human clinical trials for evaluation in PD and other age-associated neurodegenerative disorders. In this regard, early to moderate PD likely may represent the stages of disease most expected to respond to a gliptin, in the light of the demonstrated efficacy of the single GLP-1R incretin mimetics exenatide [29, 31, 32] and lixisenatide [79] in recent human clinical trials, and of exenatide in early-stage disease in a progressive PD preclinical model [80, 81].

## Conclusion

This NHP study demonstrates that oral sitagliptin doses that match doses that can be safely administered to humans [41, 43, 44] induce elevations in plasma and CSF levels of GLP-1 and GIP that align with the efficacy of sitagliptin in a well characterized model of PD in rodent [41, 43, 44]. This study thereby provides a critical translational step towards the clinical repurposing evaluation of sitagliptin in human PD and other neurodegenerative disorders for which aging is the greatest risk factor.

## Data Availability

All datasets from the current study are available from the corresponding authors on reasonable request.
